# Environmental, urinary iodine status and prevalence of goitre among schoolchildren in a high altitude area of Saudi Arabia

**DOI:** 10.12669/pjms.312.6637

**Published:** 2015

**Authors:** Mohamed Salah Omar, Dalia El-Sayed Desouky

**Affiliations:** 1 Mohamed Salah Omar, Chemistry Department, Faculty of Science, Benha University, Benha, Qalyubia, Egypt. Division of Biochemistry, Department of Pharmacology, College of Clinical Pharmacy, Taif University, Al-Haweiah, Taif 21974, Saudi Arabia; 2 Dalia El-Sayed Desouky, Department of Public Health and Community Medicine, Faculty of Medicine, Menoufyia University, Shebin El-Kom, Egypt. Department of Public Health and Community Medicine, Faculty of Medicine, Taif University, Al-Haweiah, Taif 21974, Saudi Arabia

**Keywords:** Iodine deficiency, Environment, Goitre prevalence, Thyroid hormones

## Abstract

**Objective:**

This study aimed to assess the iodine deficiency, prevalence of goitre among schoolchildren and measuring environmental iodine in Taif city, Saudi Arabia.

**Methods:**

A cross-sectional multistage cluster-sampling methodology was done on 1887 schoolchildren. Their urinary iodine concentration (UIC) and goitre prevalence was assessed. Blood level of triiodothyronin, thyroxine and thyroid-stimulating-hormone was carried out for students with normal, mild; moderate and sever iodine deficiency. The iodine content of salt, water and soil was also assessed.

**Results:**

Goitre prevalence was 7.4% and about 71% of the participants had UIC less than 100μg/L. An inverse relationship between median UIC and the percent prevalence of goitre was found. The mean serum T3, T4 and TSH were 1.05ng/dL, 6.81µg/dL, and 5.69mIU/L, respectively. A significant positive correlation was found between the mean value of urinary iodine and the mean value of both T3 and T4. While a significant negative correlation between the mean value of urinary iodine and the mean value of TSH was also noted.

**Conclusions:**

The results revealed the presence of a potential public health problem of iodine deficiency among school going children in high altitude areas of Saudi Arabia. There is a need to monitor and evaluate the salt iodization process, and distribute adequately iodized salt in the affected areas.

## INTRODUCTION

Iodine deficiency disorders (IDDs) is the most common cause of preventable brain damage, mental retardation and stunted growth in children.^1^ More than 90% of iodine intake is excreted in urine, thus urinary iodine excretion (UIE) is considered a good biochemical marker of recent dietary intake of iodine.^2^ Thyroid hormones are essential for growth and development of the brain and central nervous system and the regulation of important metabolic processes.^3^ Enlargement of thyroid gland is the common manifestation of iodine deficiency.^4^ Schoolchildren (6 to 12 years) are the most vulnerable group to iodine deficiency.^5^ Its manifestations can range from minor neurological changes, to impaired learning ability and underperformance in school.^2^ Iodine deficiency in children can be assessed by determination of urinary iodine concentration (UIC), the assessment of size of the thyroid gland^6^ and the determination of T3, T4 and TSH concentration in blood.^7^ In Saudi Arabia; a cross-sectional national epidemiological survey was conducted in 1997 to study the iodine status among schoolchildren. The southern province had the highest percentage of subjects with low UIC.^8^ Another study was done in Asir region in 2001 on schoolchildren which revealed a high prevalence of goitre among the population living in high altitude areas.^9^

Soils in mountains are poor in iodine content, thus food grown on this soil is also very low.^8^,^10^ Iodine concentration in salt, water and soil drawn from such areas is an index of human’s natural iodine intake.^11^ Taif city is a high altitude area located more than 2400 meter above sea level. An IDD control program using the universal salt iodization (USI) strategy started in Saudi Arabia in 1997.^8^ The aim of this study was to assess the status of iodine deficiency and the prevalence of goitre among schoolchildren in Taif city.

## METHODS

A cross-sectional study on a sample of schoolchildren aged 6-14 years was carried out during the period from February to June 2013. The study setting was primary and preparatory schools in Taif City.

### Population and sampling

Population of the study was students (6-14 years). Multistage sampling methodology was carried out. Following simple random sampling technique, ten schools were randomly selected from all primary and preparatory schools. One class from each grade was randomly chosen following the same sampling technique. The total number of children registered in the selected classes (2012/2013 academic year) was 2564. Students who abstained from the study, didn’t bring the consent or remained absent during the study days were excluded. After exclusion, a total sample of 1887 schoolchildren from the selected ten schools constituted the study participants. Of the participants, 793 students were females (42%) and 1094 were males (58%).

### Iodine deficiency indicators

Indicators used to evaluate the iodine nutritional status were UIE, thyroid size by palpation, iodine content in edible salt samples, TSH and thyroid hormones concentration in blood.^6^ All the participants were subjected to: (a) Personal interview to collect data about age, gender and school level, (b) Local examination of the neck to assess the thyroid size which was carried out by an experienced endocrinologist to minimize the risk of inter-observer variation. Goitre classification was done according to criteria recommended by the Joint WHO/UNICEF/ICCIDD Consultation.^6^

### Biochemical analysis

Determination of urinary iodine concentration: A random urine sample was obtained from each child in a clean urine cups and kept in frozen at -70oC until assayed for urinary iodine (child and school number, and date of urine collection were recorded on every bottle). The level of urinary iodine was determined using the Sandell-Kolthoff reaction.^6^ The WHO cut-off points for urinary iodine levels were used to define iodine deficiency as well as to classify the severity of the deficiency: optimal UIC >99µg/L; mild 50–99µg/L; moderate 20-49µg/L; and severe UIC deficiency < 20µg/L.^6^

### Measuring of T3, T4 and TSH

Based on the values of UIC obtained and after exclusion of non-respondents, a systematic random sampling methodology was carried out and blood samples were taken from 104, 150, 104 and 61 students with normal, mild; moderate and sever UIC, respectively. The collected blood sample was incubated for an hour at room temperature and the unclotted portion of the blood was centrifuged at 2000 rpm for 20 minutes. The principle of the assay is Electrochemiluminescence (ELC) immuneassay, using Roche Cobas kits following manufacture’s instructions.^12^ The normal reference ranges of total T4, T3 and total TSH were 4.5-12.6µg/dL, 0.39-1.37ng/dL and 0.3- 4mIU/L, respectively.^13^

### Determination of the environmental iodine status

1- **Iodine in water:** Twenty samples of drinking water were collected from schools located in north, south, east and west Taif city. Samples were collected in screw capped polyethylene bottles washed with distilled water. All samples were kept at 4°C, and iodine content was measured using the Karmarkar method.^14^
2- **Iodine in salt:** All the studied students were asked to bring salt samples consumed in their homes in air-tight polyethylene containers to measure its iodine content. We were able to collect 1853 salt samples, where students were asked to bring them in a tightly sealed plastic pouch and stored at room temperature till analysis. Salt iodine content was measured using the iodometric titration method^15^ The results were expressed in parts per million (ppm) and iodine concentration was recorded as <15, and >15ppm.^16^ Salt samples having iodine content less than 15ppm were classified as with inadequate iodine.^17^
3- **Iodine in soil:** Samples from superficial soil were collected from different areas (north, south, east and west) in Taif city. Soil samples were collected to determine the iodine content using the arsenic-cerium redox method.^18^ The reaction indicator and seconds-counter were used to indicate reaction end point and time. As there was a linear correlation between the logarithm of iodine concentration and the logarithm of reaction time, iodine concentration in solution could be calculated by double logarithmic regression equation. Soil samples were incinerated at 550°C for 4 hours with mixed alkalinous compounding chemicals including potassium carbonate, zinc sulfate, potassium chlorate and sodium chloride. The ash left was dissolved in distilled water and the supernatant was removed for detection.

### Administrative aspects and ethical considerations

Official approvals of the scientific research committee of Taif University, and from the General Director of basic education of Taif governorate were obtained. A written consent was signed by the student parents before sharing in this study.

### Statistical analysis

Data were analyzed by the SPSS version 16. The quantitative data was expressed as mean, median and standard deviation (Mean ±SD) and analyzed by Student t-test, and for the non-normally distributed variables the Mann Whitney test was used. The Pearson correlation test was applied to test the relationship between variables. Qualitative data were expressed as number and percentage and analyzed by applying Chi-square. p values less than 0.05 were considered significant.

## RESULTS

**Fig.1:**
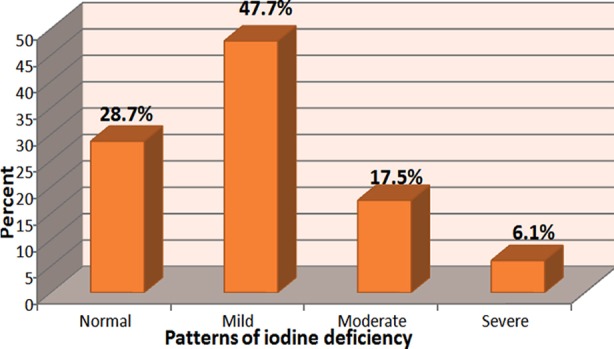
Classification of the studied participants according to different patterns of iodine deficiency (n=1887)

**Fig.2:**
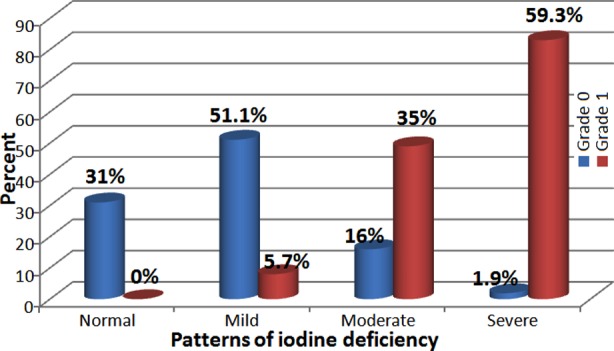
Distribution of goitre grades regarding different patterns of iodine deficiency. Chi square (χ^2^): 820.7 p-value: <0.05

A total of 1887 schoolchildren, aged 6-14 years were studied (58%male and 42% female, 70.4% in primary schools and 29.7% in preparatory schools. The median level of UIC was 84µg/L. Of them, 47.7%, 17.5% and 6.1% had mild, moderate and severe iodine deficiency, respectively (Fig.1). Only 7.4% of the studied group had goitre grade 1 and no one was found to have goitre grade 2 (Table-I). A significant difference was found between females and males according to goitre prevalence and a significant difference was found between the two age groups (below and above ten years) according to goitre grades. Participants with grade 1 goitre had a significantly lower median UIC (17.5µg/L), compared to those with grade 0 goitre (86µg/L)(P<0.05). In addition; there was a significant difference between the two goitre grades according to different patterns of iodine deficiency (Fig.2). The mean serum T3, T4 and TSH was 1.05ng/dL, 6.81µg/dL, and 5.69mIU/L, respectively. A significant positive correlation was found between the mean value of UIC and the mean value of T3 and T4 and a significant negative correlation between the mean value of UIC and the mean value of TSH (Table-III). The median T4 value for children having goitre grade 0 was 5.29μg/dL, compared to 4.15μg/dL for those with goitre grade 1. On the other hand, the mean value of TSH in participants with goitre grade 0 was 5.6mIU/L, compared to 5.9mIU/L in those with grade 1 goitre (P<0.05) (Table-IV). The mean value of iodine content of salt samples was 18.1ppm, and 61.6% of samples had iodine content ≥15ppm. The mean water iodine content was 4.6μg/L, and the mean iodine content of the collected soil samples was 0.04mg/Kg.

**Table-I:** Distribution of goitre grades according to clinical examination among the participants regarding their age and sex.

Parameter	Goitre grade				χ^2^	p-value
	Grade 0 (No. 1747)		Grade 1 (No. 140)			
	No.	%	No.	%		
*Age/ years*						
≤10	1021	93.6	69	6.4	4.46	<0.05
>10	726	91	71	9		
*Sex*						
Female	713	40.8	80	57.1	14.19	<0.05
Male	1034	59.2	60	42.9		

**Table-II:** Distribution of goitre grades regarding urinary iodine level.

Parameter	Goitre grade		Mann-Witney Test	p-value
	Grade 0 (n=1747)	Grade 1 (n=140)		
	Median, range	Median, range		
UI (µg/L)	86, 181	17.5, 85	18.23	<0.05

**Table-III:** Correlation between urinary iodine and T3, T4 and TSH.

Hormone	Urinary iodine	
	R	P value
T3 (ng/dL)	+0.097	<0.05
T4 (µg/dL)	+0.460	<0.05
TSH (mIU/L)	−0.279	<0.05

**Table-IV:** Distribution of goitre grades regarding the mean value of thyroid hormones level among the participants.

Parameter	Goitre grade		t-test	p-value
	Grade 0 (n=366)	Grade 1 (n=53)				
	Mean±SD	Mean±SD		
T3(ng/dL)	0.9±0.3	1.1±0.3	3.14	<0.05
TSH (mIU/L)	5.6±0.6	5.9±0.6	4.61	<0.05

N.B. For T4 comparison between groups, z test of Mann-Whitney was 5.06 and p-value was <0.05. (Median T4 value for grade 0 was 5.29μg/dL and their range was 8.6μg/dL. The median T4 goitre grade 1 was 4.15μg/dL and their range was 8.22μg/dL).

## DISCUSSION

In the present work, it was observed that the majority of the children enrolled in this study had UIC less than 100μg/L (Fig.1). The median level of UI observed in this study was lower than that found in an earlier Saudi cross-sectional national epidemiological survey, where median UIC was 110μg/L.^8^ The observed low median UI in this study could be attributed to a number of factors based on the geological and geographical location of Taif city as being a high altitude mountainous area. Soil on the mountains are poor in iodine due to the effect of heavy rains, iodine in these areas is swept away and replaced by new soil made of iodine poor crystalline rocks.^19^

Moreover the usual presence of iodine in the top layer of the soil at high altitudes areas tend to be easily leached away by erosion, this explains the poor iodine content in crops grown in these iodine deficient soils.^20^ Being away from the sea, population of the Taif city has a decreased access to sea foods which makes residents susceptible to iodine deficiency which was found in previous studies.^18^ In this study, the significant higher goiter prevalence among female students compared to males was found in another study.^21^ This may be due to differences in iodine metabolism during the growth phases of girls and boys.^22^ The significant difference between the two age groups (below and above 10 years) according to goitre grades, was observed in other studies.^23^ The significant difference observed between goitre grades (0 and 1) according to the mean value of UIC and the different patterns of iodine deficiency (Table-II, Fig.2), was in agreement with other studies.24 The WHO guidelines on goitre suggest that a total goitre rate of 5% or more in schoolchildren indicates the presence of a public health problem.^2^

In the present study 7.4% students had goitre grade 1 (Table-II). This finding is somewhat in agreement with others, particularly the one done in Riyadh city, where goitre prevalence was 8%.^24^ However, the goitre rate in this study was higher than that observed in a study done in Gizan city, where the prevalence of goitre grade 1 was 4%.^8^ The higher goitre prevalence in Taif city as compared to other areas in Saudi Arabia, and it could be attributed to the geological and geographical factors of the city being a high altitude and mountainous area.^11^ In addition, it is known that altitude related hypoxia stimulates the thyroid function, so the effect of iodine deficiency could result in more exaggerated thyroid hyperplasia.^25^ Iodine-deficient populations have higher serum TSH concentrations and lower serum T4 concentration, as the pituitary gland secretes TSH in response to circulating levels of T4, and the serum TSH rises when serum T4 concentrations are low.^26^ The mean serum T3 was found to be in a high normal level, the mean serum T4 was found to be in a low normal level, and the mean serum TSH was found to be in a high level.^13^ This may be explained by thyroidal secretion of T4 and T3 in the proportion in which they exist within the gland, preferential secretion of T3, or increased peripheral conversion of T4 to T3. The thyroid gland produces T3 more than T4 selectively when it encounters a relatively severe iodine-deficiency. The tendency to increase T3 secretion and increase serum T3:T4 ratio is important in the adaptation to iodine deficiency as T3 possesses about 4 times the metabolic potency of T4, but requires only 75% as much iodine for synthesis.^27^ The significant positive correlation found between the mean value of UI and the mean value of T4, and the significant negative correlation found between the mean value of UI and the mean value of TSH (Table-III) are in agreement with other studies.^28^ The significant difference found between the goitre grades according to the mean value of T3 and TSH and the median value of T4 (Table-IV) was in agreement with other studies which showed that children with simple goitre had decreased T4 production and increased TSH level.^29^

Only 61.6% of studied salt samples had iodine content (≥15ppm). This is lower than the recommended level as the WHO/UNICEF/and ICCIDD recommend that 90% of household salts should be iodized at a level of 15ppm.^30^ The iodine content of fresh water can range from 1-50μg/L.^31^ In the present study; iodine content of water samples was considered low. A result that was obtained from a Saudi study which analyzed iodine content of tap water and drinking mineral water from different regions of the country.^32^

No standard measurements of iodine in water exist because iodine concentrations vary across the world. According to the Chinese standards that define water iodine value less than 10μg/L as iodine-deficient district, and according to an Indian study where iodine content of drinking water in goitrous areas ranged from 3-16μg/L as compared to 5-64μg/L in non-goitrous areas, iodine content in Saudi water is still found low.^18^,^31^ The iodine deficiency in an area is mainly judged from iodine content of soil, level of iodine in drinking water and iodine content of food grown in that soil.^33^ In this study, the iodine content of the collected soil samples was considered low as proofed by a previous Saudi study which revealed an overall low iodide content in the soil of Saudi Arabia.^32^

## CONCLUSION

The present study showed that iodine deficiency among Saudi students in Taif city is a potential public health problem. Goitre prevalence was 7.4% and about 71% of the participants had UIC less than 100μg/L. A significant positive correlation was found between the mean value of UI and the mean value of both T3 and T4. At the same time a significant negative correlation between the mean value of UI and the mean value of TSH. Considering the study results, there is a need for increasing promotion of iodized salts to Taif city, with paying great attention to school students through an nutritional intervention program. Incorporation of health education massages about iodine deficiency in school curriculum will ensure increase in long-term awareness. Policy makers in the city should ensure a continuous monitoring and evaluation of the salt iodization process, and the distribution of adequate amounts of iodized salt to whole city.
